# Prevalence and factors associated with food insecurity in southern Mozambique

**DOI:** 10.1093/eurpub/ckac131.229

**Published:** 2022-10-25

**Authors:** E Militao, O Uthman, E Salvador, S Vinberg, G Macassa

**Affiliations:** 1 Department of Health Sciences, Mid Sweden University, Sundsvall, Sweden; 2 Department of Public Health and Sport Science, University of Gävle, Gävle, Sweden; 3 Department of Biological Sciences, Eduardo Mondlane University, Maputo, Mozambique; 4 Warwick Centre for Global Health, University of Warwick, Coventry, UK; 5 Department of Global Health, Stellenbosch University, Stellenbosch, South Africa; 6 EPI Unit, Instituto de Saúde Pública, Universidade do Porto, Porto, Portugal

## Abstract

**Background:**

Food insecurity (FI) is one of the major causes of malnutrition in low- and middle-income countries. In Mozambique, the burden of FI and how it’s related to negative health outcomes is unknown. This study aimed to investigate the prevalence of FI as well as the factors associated with FI in southern Mozambique.

**Methods:**

Preliminary data from 301 household heads residing in suburb and peri-urban districts of Maputo were analysed in a cross-sectional design. Accordingly, FI was assessed using the 8-items of the United States Department of Agriculture Household Food Security Survey Module, and its association with various factors was determined through multiple regression models.

**Results:**

The prevalence of FI was 62.8% (23.6% of households had mild FI, 16.6% had moderate FI, 22.6% had severe FI). Based on multiple regression models, 10 variables (out of 11) were relevant drivers of FI and reached statistical significance (p-value<0.05) with focus on food diversity, climate change, illnesses, household income, number of meals, type of work, household size.

**Conclusions:**

These preliminary findings suggest the need for decent work and job creation. In addition, food diversity, climate change and some relevant diseases should be taken into account in the development of public health policies designed to alleviate household food insecurity in Mozambique.

**Key messages:**

• Food insecurity in Mozambique calls for joint efforts from government, private sector, international institutions and communities.

• The basic food basket for the most vulnerable groups is encouraged as a short-term solution.

